# Utilization of the Total Error Allowance Rule to Determine the Clinical Acceptability of Complete Blood Counts in the Blood Samples Collected via Peripherally Inserted Central Catheters in Cancer Patients

**DOI:** 10.1007/s12288-024-01931-7

**Published:** 2025-01-08

**Authors:** Xiaojing Xue, Jie Tang, Yuwei Yang, Lijuan Yang, Siqi Zhang, Xiaobo Du, Gang Feng, Chao Guo, Rong Chen, Yalan Yang

**Affiliations:** 1 Department of Oncology, Mianyang Central Hospital Affiliated to School of Medicine of University of Electronic Science and Technology of China, Mianyang, 621000 PR China; 2 Department of Laboratory Medicine, Mianyang Central Hospital Affiliated to School of Medicine of University of Electronic Science and Technology of China, Mianyang, 621000 PR China

**Keywords:** Total error allowance, PICC, Blood sample, Complete blood count

## Abstract

Peripherally inserted central catheters (PICCs) are widely used in oncology patients for drug infusion and nutritional support; however, their usefulness for blood sample collection has not been clarified. This study aimed to evaluate the clinical acceptability of complete blood count (CBC) measurements in blood samples drawn from PICC lines in oncology patients. Blood samples were drawn twice successively from the PICC lines (PICC0 and PICC1 samples, respectively), and a peripheral blood collected from the contralateral limb was used as reference sample. Significant differences and sources of bias in the CBC measurements in two PICC samples were analyzed. Clinical acceptability was determined based on the total error allowance (TEa) rules. Except for hemoglobin and erythrocyte, no significant differences were observed in the other parameters between PICC1 and reference samples (all *P* > 0.05), including leukocyte, hematocrit, platelet, erythrocyte parameters and leukocyte differential counts. Deviations of erythrocyte and hemoglobin in PICC1 samples was only attributed to random error, whereas proportional bias were also observed in PICC0 samples. According to the given TEa, erythrocyte, leukocyte, hemoglobin, and platelet of the PICC1 samples met the 1/2 TEa criteria and had favorable clinical acceptability. However, the hematocrit and the three erythrocyte parameters failed to meet the 1/2 TEa criteria. Adherence to the TEa criteria may provide higher clinical acceptability for CBC results. Satisfactory results in oncology patients may be involve discarding the first 2 mL of blood samples during sample collection using PICC lines.

## Introduction

Peripherally inserted central catheters (PICCs) are silicone central venous catheters that are inserted into the superior vena cava through a peripheral vein puncture in the arm in front of the elbow. Commonly used puncture sites include the basilic, median cubital, and cephalic veins. A central venous catheter is usually recommended when the duration of systemic anti-cancer treatment (SACT) exceeds 3 months or even longer, or when no suitable peripheral vein is available [1]. PICCs are preferred for SACT patients because of their lower dead space volume and greater convenience. PICCs are directly connected to a large central vein (usually the superior vena cava), making it more suitable for the infusion of chemotherapeutic agents, antibiotics, parenteral feedings, and blood products in oncology patients. PICCs have also been clinically proven to prevent local venous damage and tissue necrosis caused by the irritative effects of anti-tumor treatments [2].

Since central venous access is created in PICCs, blood collection and drug infusion can be conveniently performed through PICCs [3]. The 8th edition of the “Infusion Therapy Standards of Practice” guideline, published in 2021 by the Infusion Nursing Society (INS), recommends collecting blood samples using short peripheral venous catheters (e.g., indwelling needles). Compared to direct venipuncture, the test results of such samples did not affect clinical decision-making. The guideline also states that PICCs and midline catheters can be used to collect blood specimens, but there is currently no technical or evidence-based support for this procedure [4]. Therefore, once it is established that blood samples can be feasibly collected using the pre-existing vascular access in PICCs; this would offer a convenient method for patient blood sampling. This approach not only addresses the challenge of difficult blood collection in patients with cancer, but also mitigates the risks of frequent punctures, reduces pain, and helps avoid unforeseen complications associated with repeated blood draws.

To ensure the availability of blood samples collected from PICC, The Society of Critical Care Medicine (SCCM) recommends collecting blood samples at least 2 min after stopping all infusions and using a flushing technique to prevent contamination from residual medications [5]. The Clinical and Laboratory Standards Institute (CLSI) recommends using the discard method to ensure blood sample quality. For non-coagulation tests, discarding twice the volume of the catheter dead space is recommended. For coagulation tests, discarding 5 mL of blood or six times the dead space volume is advised [6]. Discarding twice the dead space volume ensures the accuracy of blood cell count results in blood samples of patients with cancer [7]. Currently in China, the standard practice is to discard 5 mL of blood for adults and 3 mL for children. However, this was limited to the evaluation of basic chemical and hematological test results [8]. Hence, the current validation of sampling methods at PICC sites and their application across various diseases lacks sufficient clinical evidence. For patients requiring frequent blood collection for testing, the discard method may exacerbate iatrogenic anemia [9]. Therefore, determining the minimum amount of blood to discard to avoid wastage has become crucial in defining standards.

To date, studies of blood sampling approaches from PICCs in oncology patients have been relatively rare, resulting in a lack of evidence to validate the accuracy of the tests. The methodological differences, including random error in measurements, and individual biological variations, in laboratory tests have led in certain inherently acceptable fluctuations in the test results. Additionally, obtaining consistent results is difficult even when the same laboratory test is performed multiple times on the same sample. Although statistical methods, such as paired t-tests, Bland–Altman consistency analysis, and Passing–Bablok regression analysis, may be used to verify the comparability of laboratory test results, relying solely on these methods is inadequate for accurately assessing their clinical acceptability due to the influence of methodological differences, random measurement errors, and individual biological variations. Therefore, total error allowance (TEa) is particularly important to assess the comparability of test results [10]. TEa considers the overall error of laboratory tests, including intra-day variability, inter-day variability, and biological variation. This facilitates a proper assessment of whether the differences in test results between two different scenarios are acceptable. Therefore, the aim of this study was to investigate the differences in complete blood count (CBC) results of blood samples collected from the PICC lines of oncology patients and determine the clinical acceptability of these results based on TEa.

## Methods

### Patients

This prospective study included 40 inpatients at the Department of Oncology of Mianyang Central Hospital affiliated to the School of Medicine, University of Electronic Science and Technology of China, from March 2023 to April 2023. There were 23 males and 17 females aged 48–77 years (mean age: 64.15 ± 8.68 years). According to the ICD-11 disease classification, this study included 6, 6, 4, 4, 4, 4, 4, 3, 3, and 2 cases of cervical, gastric, esophageal, lung, liver, ovarian, nasopharynx, rectal, duodenal, and breast cancer, respectively. All patients received placement of single-lumen PICC manufactured by Bard Access Systems, INC. The specifications of the PICC were 4.0 F (1.40 mm OD) × 60 cm, with a dead space volume of 0.48 mL.

### Blood Sample Collection

Blood samples were collected sequentially in the morning from all patients in a fasting state. The samples were collected from their PICC lines and peripheral veins by one designated phlebotomists, strictly following aseptic conditions to ensure sterility. All patients were in supine position. Blood was collected from the PICC line after a cessation of infusion for at least 3 h. Using a 10-mL syringe without a needle, 2.0 mL of venous blood was withdrawn from the PICC catheter interface. This blood was then transferred into a disposable vacuum blood collection tube containing EDTA-2 K anticoagulant (BD, USA; PICC0 sample). Another 10-mL syringe without a needle was used to draw additional 2.0 mL of venous blood, which was subsequently transferred into a separate vacuum blood collection tube containing EDTA-2 K anticoagulant (PICC1 sample). The PICC puncture site was then disinfected, and the catheter was flushed and sealed as per the standardized procedure Peripheral venous blood was collected immediately after the blood collection from the PICC line. Using the vacuum collection system, 2.0 mL of venous blood was drawn from the median cubital vein of the contralateral arm. This blood was placed into a third disposable vacuum blood collection tube containing EDTA-2 K anticoagulant to serve as the reference peripheral blood control.

### Complete Blood Count

CBC was performed using a Sysmex XN-9000 blood cell analyzer (Sysmex, JPN) within 2 h of sampling. Leukocyte and its differential count was determined using hydrodynamic focusing and flow cytometry, respectively. The direct current sheath flow detection method was used to assess erythrocyte and platelet counts. Hematocrit and plateletcrit were analyzed using the cumulative pulse height method. Hemoglobin was detected using the sodium dodecyl sulfate colorimetric assay. Erythrocyte and platelet parameters, including mean corpuscular volume (MCV), mean corpuscular hemoglobin (MCH), mean corpuscular hemoglobin concentration (MCHC), red cell distribution width-standard deviation (RDW-SD), red cell distribution width-coefficient of variation (RDW-CV), mean platelet volume (MPV), platelet distribution width (PDW), and platelet larger cell ratio (P-LCR), were automatically calculated or derived from the above test results by the instrument.

### Consistency Analysis of the Test Results Between Blood Samples Collected from PICCs and the Peripheral Vein

Using reference peripheral blood as a baseline, paired t-tests were used to identify differences in various parameters of the complete blood cell count. Subsequently, a Mountain plot was used to visually depict the extent of differences between the two samples obtained sequentially from the PICC line. Finally, a Passing and Bablok regression analysis was performed to discern the sources of deviations in the differing parameters. The paired t-test provides a more rigorous test of consistency than the independent t-test and correlation analysis [11, 12]. The Mountain plot provides a visual representation of the central tendency and dispersion of the deviations [13]. The degree to which the peak value deviated from 0 illustrated the magnitude of the median deviation (i.e., central tendency), while the width of the base indicated the degree of dispersion of the deviation. Passing and Bablok regression analysis is used to determine the magnitude of systematic bias, proportional bias, and random error [14]. If the 95% confidence interval (CI) of the intercept of the regression curve excluded 0, a systematic bias was indicated. If the 95% CI of the slope of the regression curve excluded 1, a proportional bias was indicated. Concurrently, the residual standard deviation (RSD) reflects the magnitude of random error.

### Analysis of the Clinical Acceptability of the Test Results in Blood Samples Collected from PICCs and Peripheral Veins

Based the industry standard “Guidelines of venous blood specimen collection” by the National Health Commission (NHC) of China, the laboratory results from unmatched testing systems were used for comparability analysis; additionally, TEa-based accuracy assessment was adopted to analyze the clinical acceptability of blood collected from PICCs. The guidelines for comparability of laboratory results for unmatched testing systems recommend including 40 paired samples. According to the TEa criteria, for the 40 paired test samples, when the relative deviation of the test results between the measurement system and the reference system was: (i) < ± 1/2 TEa, the deviation of the measurement system was within the lowest control range; (ii) < ± 1/3 TEa, it was within the favorable control range; and (iii) < ± 1/4 TEa, it was within the optimal control range. If > 80% of the data had a relative deviation of < ± 1/2 TEa, the results of the assay system were deemed clinically acceptable. The TEa criteria were derived from the specifications by NHC, with the TEa being 6.0%, 7%, 8%, 9%, 15%, and 20% for both erythrocyte and hemoglobin, both MCV and MCH, MCHC, hematocrit, leukocyte, and platelet, respectively.

### Statistical Analysis

SPSS 22.0 (SPSS Inc., Chicago, IL, USA) and MedCalc 20.1 (MedCalc, Mariakerke, Belgium) software packages were used for the statistical analysis. All test data were determined to be normally or approximately normally distributed through the one-sample Kolmogorov–Smirmov test and Q–Q plot. The test data are presented as mean ± standard deviation. Paired t-tests were performed to analyze the result differences between the PICC samples and reference peripheral bloods. The result concordances between the PICC samples and reference peripheral bloods were compared using a Mountain plot analysis. Passing and Bablok regression analysis was performed to determine the proportional bias, systematic bias, and random error. The magnitude of deviation, the dispersion degree of the deviation, and proportional and systematic bias were indicated as mentioned previously. A Bland–Altman plot was constructed using the test results of the reference peripheral blood as the horizontal axis, and the relative deviation of the test results obtained from blood sample at the PICC insertion site compared to the reference peripheral blood test results as the vertical axis. To determine the clinical acceptability based on the TEa, ± 1/2 TEa line was drawn on the Bland–Altman plot to observe the rate of data falling outside the ± 1/2 TEa line. A test level of α = 0.05 was adopted.

## Results

### Patients

Among the 40 patients, there were more males (23, 57.5%) than females (17, 42.5%), and the most common diagnoses were cervical and stomach cancer. The mean duration of PICC was 171.07 ± 66.07 days. The relevant data are shown in Table 1.

**Table 1:** Basic patient information

	*n*	Percentage
Gender		
Male	23	57.5
Female	17	42.5
Age		
M ± SD	64.15 ± 8.68	
range	48 ~ 77	
Diagnosis		
Cervical carcinoma	6	15
Stomach Cancer	6	15
Esophageal cancer	4	10
Lung cancer	4	10
Liver Cancer	4	10
Oropharyngeal Cancer	4	10
Nasopharyngeal darcinoma	4	10
Rectal Cancer	3	7.5
Duodenal cancer	3	7.5
Breast cancer	2	5
Catheter retention time		
M ± SD	171.07 ± 66.07	
range	25 ~ 295	

### Differential Analysis of CBC in Blood Samples from the PICC Line and Parameter Results

Differences in CBC results between the two samples obtained sequentially from the PICC line were analyzed by pairing the results of PICC0 and PICC1 samples with those of the reference peripheral blood samples, respectively. Comparing PICC0 samples results with those of the reference peripheral blood samples showed that (Table 2), except for erythrocyte parameters (|t0|=0.285–1.745, *P* > 0.05) and B (|t0|=1.158, *P* = 0.255), the differences in the CBC measurements of other blood cells were significant (|t0|=2.563–49.485, *P* < 0.05). However, the differences between the results of PICC1 samples and those of the reference peripheral blood samples were not statistically significant (|t1|=0.062–1.772, *P* > 0.05) except for erythrocyte (t1 = 2.234, *P* = 0.031), hemoglobin (t1 = 2.358, *P* = 0.023) (Table 2).

**Table 2:** Differential analysis of the CBC results of the two samples obtained sequentially from the PICC line

Item	*n*	Peripheral Blood	PICC0 Sample	PICC1 Sample	t0, P0	t1, P1
Leukocyte	40	5.90 ± 4.73	5.37 ± 4.27	5.93 ± 4.77	**5.406**,** < 0.001**	0.609, 0.546
Erythrocyte	40	3.64 ± 0.78	3.07 ± 0.62	3.60 ± 0.76	**14.881**,** < 0.001**	**2.234**,** 0.031**
Hemoglobin	40	108 ± 21	92 ± 18	107 ± 21	**15.621**,** < 0.001**	**2.358**,** 0.023**
Hematocrit	40	0.338 ± 0.062	0.290 ± 0.055	0.332 ± 0.056	**17.888**,** < 0.001**	1.772, 0.084
MCV	40	94.10 ± 11.46	95.3 ± 11.0	94.8 ± 9.8	-1.745, 0.089	-0.752, 0.457
MCH	40	29.9 ± 3.7	30.1 ± 3.2	30.0 ± 3.3	-1.087, 0.284	-0.456, 0.651
MCHC	40	318 ± 14	317 ± 15	317 ± 21	0.518, 0.608	0.404, 0.688
RDW-CV	40	15.6 ± 3.0	15.4 ± 3.0	15.4 ± 2.8	1.226, 0.227	1.453, 0.154
RDW-SD	40	53.6 ± 12.1	53.7 ± 12.2	53.8 ± 12.0	-0.285, 0.777	-0.553, 0.583
Platelet	40	172 ± 84	145 ± 64	171 ± 81	**7.331**,** < 0.001**	-0.281, 0.780
PCT	38	0.19 ± 0.08	0.16 ± 0.06	0.18 ± 0.07	**7.576**,** < 0.001**	-0.062, 0.951
MPV	38	10.6 ± 1.2	10.9 ± 1.1	10.8 ± 1.4	**-49.485**,** < 0.001**	-1.038, 0.306
PDW	38	12.4 ± 2.7	13.2 ± 3.0	13.2 ± 3.5	**-5.017**,** < 0.001**	-1.491, 0.144
P-LCR	38	29.5 ± 9.3	30.9 ± 9.2	31.1 ± 10.4	**-3.310**,** 0.002**	-1.203, 0.236

Note: MCV, MCH, MCHC, RDW-CV and RDW-SD are erythrocyte parameters. PCT, MPV, PDW and P-LCR are platelet parameters. (t0, P0) and (t1, P1) are the results of the paired t-tests for CBC when comparing PICC0 and PICC1 samples with the reference peripheral blood samples, respectively. The reduction in the number of cases of PLT-related indices by two cases was due to the absence of instrumental measurement results. The results indicated that there were no significant differences in CBC between PICC1 samples and peripheral blood, except for RBC and hemoglobin levels. However, there were notable variations observed between PICC0 samples and peripheral blood, except for RBC parameters

**Fig. 1:**
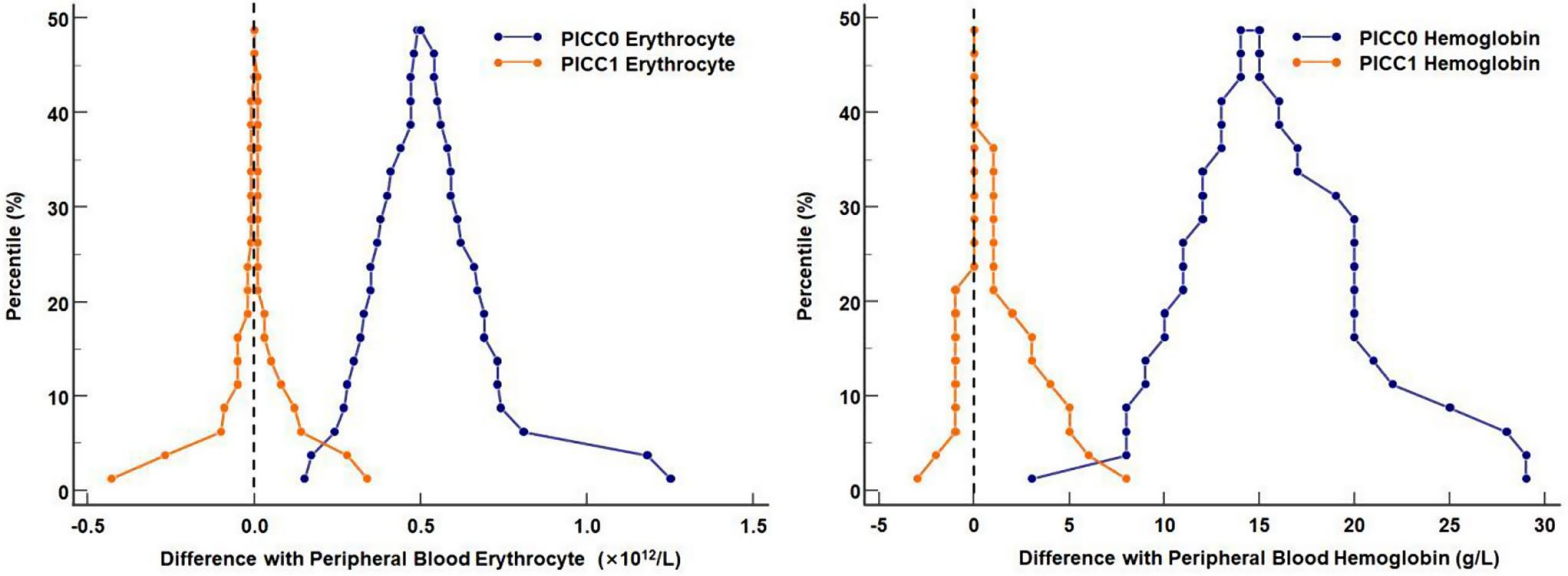
Distribution of deviations of erythrocyte and hemoglobin in the two samples obtained sequentially from the PICC line. Note: Compared with PICC0, the peak values of erythrocyte and hemoglobin in PICC1 are closer to or equal to 0, and the base widths are narrower. This indicates that the test results of PICC1 samples are closer to the test results of peripheral bloods

Mountain plots were used to analyze the strengths and weaknesses of erythrocyte and hemoglobin testing in the two samples obtained sequentially from the PICC line. Using the peripheral blood as a reference, the deviations of the peak values of erythrocyte and hemoglobin in the PICC1 samples were close or equal to 0, whereas those in the PICC0 samples were farther from 0 (Fig. 1). Moreover, the width of the base of the deviations in erythrocyte and hemoglobin of the PICC1 samples was smaller than that of the PICC0 samples. This suggested that the CBC results of the PICC1 were more accurate than those of PICC0. Compared with peripheral blood, no differences were observed in leukocyte, hematocrit, and platelet counts and the related indicators in PICC1, whereas further analysis was required to determine the source of differences in erythrocyte and hemoglobin.

### Deviation of Erythrocyte and Hemoglobin in the Two Samples Obtained Sequentially from the PICC Line

**Fig. 2:**
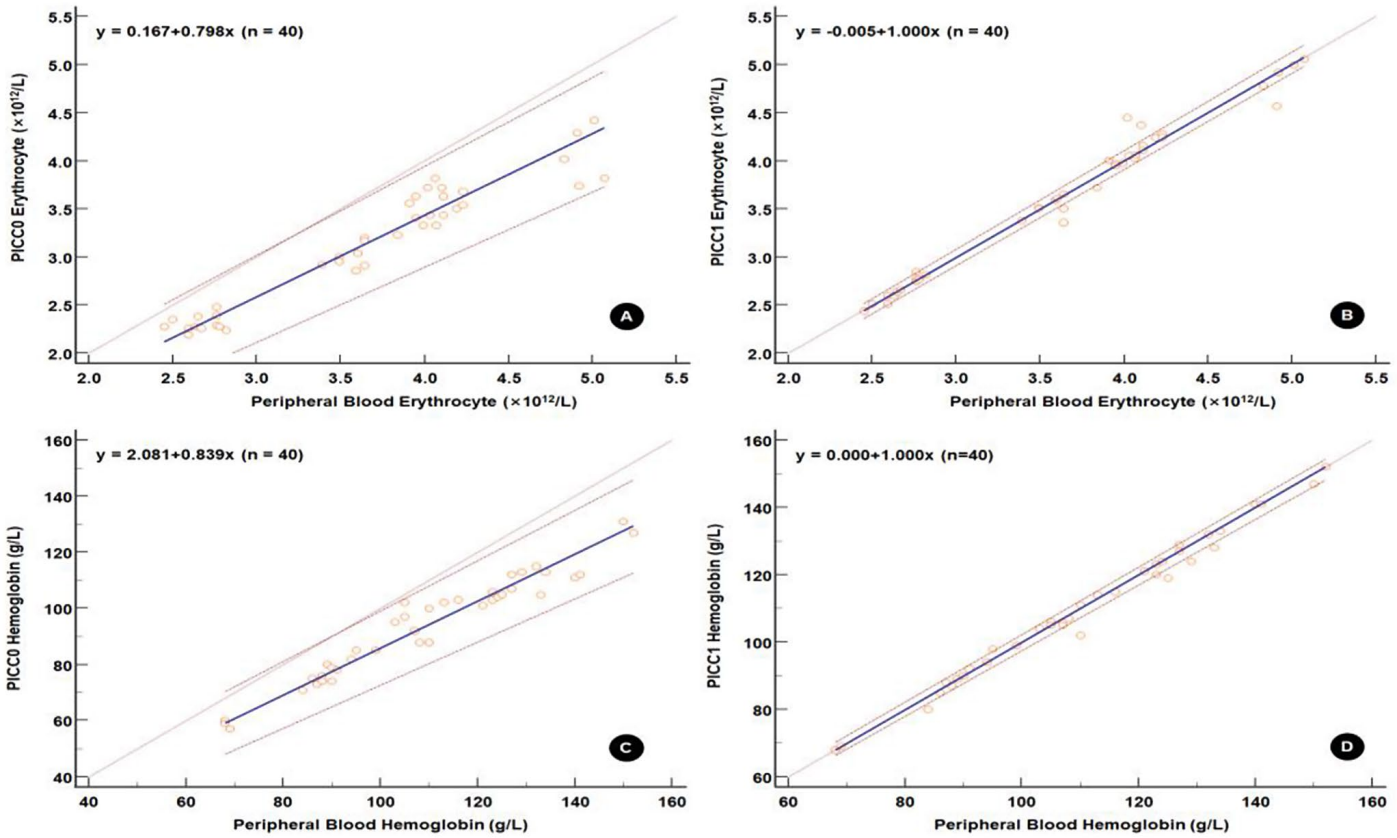
Passing and Bablok regression plot of the sources of error of erythrocyte and hemoglobin in the two samples obtained sequentially from the PICC line. Note: Passing and Bablok regression is a linear regression curve constructed by calculating the residuals perpendicular to the regression line (i.e., vertical residuals). The area between the red lines indicates the 95% CI of the regression curve

Passing and Bablok regression analysis was performed to analyze the source of differences in erythrocyte and hemoglobin of PICC0 and PICC1 samples using the peripheral blood samples as references. For the Passing and Bablok regression lines for erythrocyte and hemoglobin between the PICC1 and reference peripheral blood samples, the 95% CIs for the intercept included 0, indicating no systematic bias (Fig. 2; Table 3). Additionally, the 95% CIs for the slope included 1, indicating no proportional bias. Conversely, for the Passing and Bablok regression line for erythrocyte and hemoglobin between the PICC0 and reference blood samples, the 95% CI of the intercept included 0, indicating no systematic bias. However, the 95% CI of the slope excluded 1, indicating the presence of proportional bias. Moreover, the RSD of the linear regression of PICC1 against reference peripheral blood samples was lower compared to that of PICC0, indicating that PICC1 also had a lower random error, thereby suggesting a better consistency of the test results between PICC1 and the reference samples.

**Table 3:** Sources of error in erythrocyte and hemoglobin in the two samples obtained sequentially from the PICC line

Sources of error	Statistical indicators	PICC0		PICC1	
		Erythrocyte (×10^12^/L)	Hemoglobin (g/L)	Erythrocyte (×10^12^/L)	Hemoglobin (g/L)
Systematic deviation	Intercept (95% CI)	0.038 (–0.232, 0.241)	2.300 (–4.200, 9.077)	–0.027 (–0.088 0.000)	0.000 (–0.000, 2.300)
Proportional bias	Slope (95% CI)	0.849 (0.782, 0.925)	0.836 (0.769, 0.900)	1.007 (0.999, 1.027)	1.000 (0.975, 1.000)
Random error	RSD (± 1.96RSD)	0.177 (–0.348, 0.348)	4.291 (–8.410, 8.410)	0.118 (–0.231 0.231)	2.379 (–4.662, 4.662)
Linear validation	Cusum test	*P* = 0.97	*P* = 0.53	*P* = 0.53	*P* = 0.70

Note: RSD, residual standard deviation, is a measure of the random difference in test results between two blood samples. A larger ± 1.96RSD interval indicates poorer consistency of the test results between the two blood samples. The Cusum test is employed to assess the goodness of fit between a linear model and data. A P-value less than 0.05 suggests the absence of a linear relationship between the two measured values

### Clinical Acceptance of Erythrocyte and Hemoglobin Results Based on TEa

**Fig. 3:**
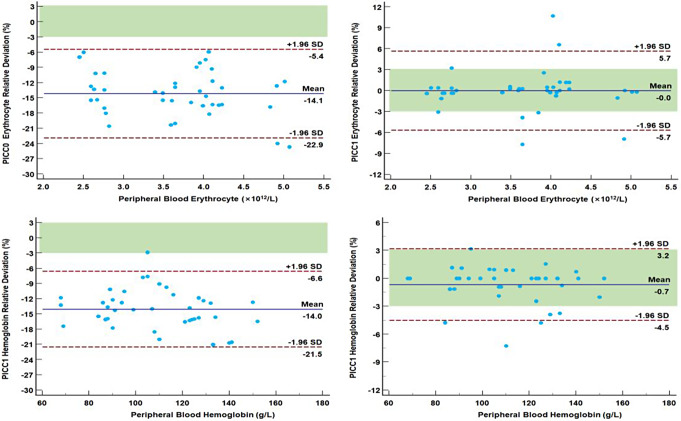
Relative deviation of erythrocyte and hemoglobin of the two samples obtained sequentially from the PICC line. Note: The green area indicates the minimum controllable range for the relative deviation based on TEa, i.e., ± 1/2 TEa area. The TEa criterion is derived from the NHC specifications, which is 6.0% for both erythrocyte and hemoglobin

The Bland–Altman plot of the relative deviation of erythrocyte and hemoglobin was constructed using the relative deviation of the test results between the PICC and reference peripheral blood samples as an observation indicator. The ± 1/2 TEa area was added to the plot. In PICC0 samples, 40/40 (100%) and only 1/40 (2.5%) of the relative deviation of erythrocyte and Hemoglobin fell within the ± 1/2 TEa area, respectively (Fig. 3). For PICC1, 32/40 (80.0%) and 34/40 (85.0%) of the relative deviation of erythrocyte and Hemoglobin fell within the ± 1/2 TEa area, respectively. Overall, the results of both erythrocyte and Hemoglobin of PICC1 met the minimum standards for clinical acceptance.

The same approach was adopted to analyze the other six tests with TEa criteria. We found that 33/40 (82.5%) of the relative deviation of leukocyte and platelet between PICC1 and peripheral blood samples fell within the ± 1/2 TEa area (Table 4), which indicated that it met the minimum criteria for clinical acceptance.

**Table 4:** Distribution of relative bias [n (%)] for the other 6 tests and the clinical acceptance

Item	Relative deviation between PICC0 samples and peripheral bloods			Relative deviation between PICC1 samples and peripheral bloods		
	Within ± 1/2TEa	Outside ± 1/2TEa	Clinical acceptance	Within ± 1/2TEa	Outside ± 1/2TEa	Clinical acceptance
Leukocyte	21(52.5)	19(47.5)	NA	32(80.0)	8(20.0)	Acceptable
Hematocrit	0(0.0)	40(100.0)	NA	18(45.0)	22(55.0)	NA
MCV	0(0.0)	40(100.0)	NA	19(47.5)	21(52.5)	NA
MCH	25(62.5)	15(37.5)	NA	28(70.0)	12(30.0)	NA
MCHC	26(65.0)	14(35.0)	NA	30(75.0)	10(25.0)	NA
Platelet	15(37.5)	25(62.5)	NA	32(80.0)	8(20.0)	Acceptable

Note: MCV, MCH and MCHC are three of erythrocyte parameters. NA, not acceptable

## Discussion

Collecting blood samples from PICC lines may reduce patient discomfort, increase medical safety for professionals, and reduce the risk of accidental percutaneous needlestick injuries [7]. In clinical practice, nurses may collect blood from the PICC line, an already existing catheter, due to the poor peripheral vascular conditions in patients. However, the acceptability of this method of blood sample collection has not been clinically studied to date.

Blood collected from PICC lines can be used to monitor various antibiotic concentrations accurately [15]. Furthermore, the prothrombin time, partial thromboplastin time, and fibrinogen in coagulation function differed from the corresponding results of the peripheral blood specimens. However, such differences did not affect clinical decision-making [16]. The results of our study showed a statistically significant difference in erythrocyte count and hemoglobin in blood samples collected from PICC for CBC test. However, based on the TEa rules for assessment, the leukocyte count, neutrophil count, erythrocyte count, platelet count, and hemoglobin test results were clinically acceptable. Moreover, these five parameters are the most crucial ones that clinicians consider when determining whether tumor treatment induces bone marrow suppression or infection risk in patients, thereby providing a foundation for subsequent treatment decisions.

One of the most important factors affecting the results of blood tests using the samples collected from PICC lines is the effect of drug residues in the tubing. Ponticelli E et al. [6] recommended the use of two operational methods, push-pull and discard method, to eliminate this effect. For the latter, studies on the discarded volumes are limited. The INS guidelines suggest 2–25 mL of blood to be discarded [4], and the CLSI recommends discarding twice the volume of the dead space for noncoagulation tests [8] and 5 mL or 6 times the volume of the dead space for coagulation tests. However, this has not been conclusive to date [17–20]. Furthermore, the results of blood collection after discarding at least 20 mL of blood were credible, which undoubtedly increases the risk of iatrogenic anemia [21–22]. Failure to discard the initial aliquots of blood drawn from PICC had a significant impact on clinical decision making and is, therefore, not recommended. The minimal amount of blood that could be accurately discarded in the operation method designed in our experiment was 2 mL (equivalent to 4 times the volume of the dead space). This material could be used to obtain a high level of clinical acceptance of the CBC results, which are the key concern for oncology patients. Moreover, such a method was less likely to cause iatrogenic anemia in patients.

The use of PICCs provided an opportunity for convenient blood collection, but increased the risk of pre-analytical errors. Therefore, quality control of the blood collected from PICC lines and assessment of the accuracy of test results are necessary. However, the physiological difference of the laboratory assays cannot be reflected through the analysis of variance alone, which is problematic [23]. In this study, the criteria for determining the clinical acceptability of the test results based on TEa was adopted to analyze the clinical acceptability of CBC measurements in samples collected from PICC lines. This study showed that except for hematocrit, all the instrumental measurements (i.e., leukocyte, erythrocyte, hemoglobin, and platelet) met the 1/2 TEa criteria and had favorable clinical acceptability. In other CBC results derived from calculations, there were no statistically significant differences in almost all erythrocyte parameters and leukocyte differential counts (except for eosinophil count), and these parameters may be clinically accepted. These findings have important implications for patients with cancer receiving long-term treatment.

Additionally, although this study found no differences in the calculated erythrocyte parameters in paired t-tests, none of the three mean erythrocyte parameters were clinically acceptable when using the 1/2 TEa criteria. Although statistical analysis to compare paired t-test differences may be performed to determine the consistency of results, the impact of non-methodological factors, such as biological variation, should also be considered when deciding on the clinical acceptance of test results. Therefore, it is the quality specifications (e.g., the 1/2 TEa criteria in this study) for medical test indicators that ultimately determine the clinical acceptability of test results. Unfortunately, only 8 of the 19 CBC items investigated in this study had clear TEa specifications. There is a long way ahead to improve the quality specifications of remaining medical test items.

PICC is an important “lifeline” for oncology patients, and violation of operation protocols in the use and maintenance of PICC lines can pose risks, such as potential contamination in the catheter lumen and issues with catheter patency [24]. Therefore, in the present study, mandatory requirements were strictly followed during blood collection from the PICC lines, i.e. stopping the use of the PICC line for at least 3 h before sampling, flushing the PICC line without injecting any fluid, wiping the positive pressure connector using alcohol cotton pads, drawing precisely 2 mL of blood perpendicular to the puncture point during sampling, and flushing the lumen with 0.9% of saline and 6–8 mL of sodium heparin before sealing the PICC line under positive pressure after sampling. Strict adherence to the operation protocol ensured the proper use and maintenance of the PICC lines. No risk of adverse effects were observed during this study.

Limitations: Despite the identification of the suitability of PICC blood sampling for detecting key blood cell count parameters of significant concern in patients with cancer, the observed unacceptability in certain parameters, including hematocrit, erythrocyte and platelet parameter measurements could restrict its applicability for complete blood cell count assessment in patients with other diseases.

## Data Availability

The raw data can be obtained on request from the corresponding author.

## Abbreviations

CBC: Complete Blood Counts
MCH: Mean corpuscular hemoglobin
MCHC: Mean corpuscular hemoglobin concentration
MCV: Mean corpuscular volume
MPV: Mean platelet volume
NHC: National Health Commission
PDW: Platelet distribution width
PICC: Peripherally Inserted Central Catheter
P-LCR: Platelet larger cell ratio
RDW-CV: Red cell distribution width-coefficient of variation
RDW-SD: Red cell distribution width-standard deviation
TEa: Total error allowance
